# Supplementary material for: Predictive and Diagnostic Values of Systemic Inflammatory Indices in Bronchopulmonary Dysplasia
Source: Children (Basel). 2023 Dec 25;11(1):24. doi: 10.3390/children11010024 (PMC10814477; doi:10.3390/children11010024)
Supplement: Supplementary file 1 [file children-11-00024-s001.zip › children-2748456-supplementary.pdf]

Table S1. Systemic inflammatory indices of preterm infants with and without BPD at birth.

At birth	Non-BPD （n = 72）	BPD （n = 50）	P
N[a]	3.46 （2.43,5.78）	3.20（1.78,7.87）	0.694
NLR[a]	1.07 （0.65,1.60）	1.17 （0.56,2.61）	0.525
PLR[a]	66.38 （52.17,85.87）	71.37 （46.77,100.01）	0.509
MLR[a]	0.18 （0.10,0.31）	0.18 （0.12,0.35）	0.446
SII[a]	253.76 （157.86,394.77）	218.69 （128.93,671.23）	0.921
SIRI[a]	0.66 （0.30,1.45）	0.59 （0.24,3.34）	0.515
PIV[a]	128.46 （68.61，352.32）	148.5 （52.12,569.04）	0.767

BPD, bronchopulmonary dysplasia; N, neutrophil count; NLR, neutrophil–lymphocyte ratio; PLR, platelet–lymphocyte ratio; MLR, monocyte–lymphocyte ratio; SII, systemic immune-inflammation index; SIRI, systemic inflammation response index; PIV, pan-immune-inflammation value. [a] Median (quartile) [M (P25, P75)]. * $P < 0.05$.

Table S2. Systemic inflammatory indices of infants with and without BPD at 72 h after birth.

72 h	Non-BPD （n = 72）	BPD （n = 50）	P
N[a]	4.20 （2.64, 6.66）	5.49 （2.67, 9.68）	0.169
NLR[a]	1.24 （0.85, 1.87）	1.76 （1.08, 3.54）	0.004*
PLR[a]	56.50 （37.03， 8.52）	54.77 （34.27， 82.99）	0.787
MLR[a]	0.39 （0.28, 0.54）	0.40 （0.27, 0.56）	0.759
SII[a]	199.44 （135.77， 389.38）	281.94 （149.24, 457.39）	0.300
SIRI[a]	1.48 （0.86， 3.02）	2.03 （0.94, 4.03）	0.259
PIV[a]	241.11 （122.85, 580.35）	223.03 （98.10, 681.09）	0.868

BPD, bronchopulmonary dysplasia; N, neutrophil count; NLR, neutrophil–lymphocyte ratio; PLR, platelet–lymphocyte ratio; MLR, monocyte–lymphocyte ratio; SII, systemic immune-inflammation index; SIRI, systemic inflammation response index; PIV, pan-immune-inflammation value. [a] Median (quartile) [M (P25, P75)]. * $P < 0.05$.

Table S3. Systemic inflammatory indices of preterm infants with and without BPD at 1 week after birth.

1 week	Non-BPD （n = 72）	BPD （n = 50）	P
N[a]	5.80 （4.00， 0.81）	7.68 （4.50, 11.57）	0.175
NLR[a]	1.29 （0.81, 2.01）	1.85 （1.01, 2.65）	0.025*
PLR[a]	52.63 （35.96, 69.11）	45.06 （30.08, 64.33）	0.213
MLR[a]	0.40 （0.31， 0.60）	0.50 （0.37, 0.73）	0.080
SII[a]	275.35 （171.40, 509.55）	327.32 （192.85, 595.12）	0.492
SIRI[a]	2.52 （1.35, 5.23）	3.48 （2.02, 6.24）	0.055
PIV[a]	542.55 （304.64, 1209.76）	803.26 （361.78， 325.25）	0.466

BPD, bronchopulmonary dysplasia; N, neutrophil count; NLR, neutrophil–lymphocyte ratio; PLR, platelet–lymphocyte ratio; MLR, monocyte–lymphocyte ratio; SII, systemic immune-inflammation index; SIRI, systemic inflammation response index; PIV, pan-immune-inflammation value. [a] Median (quartile) [M (P25, P75)]. * $P < 0.05$.

Table S4. Systemic inflammatory indices of preterm infants with and without BPD group at 2 weeks after birth.

2 weeks	Non-BPD （n = 72）	BPD （n = 50）	P
N[a]	6.76 （3,34， 8.93）	6.57 （4.72， 0.82）	0.136
NLR[a]	1.29 （0.73, 2.09）	1.46 （1.03, 2.27）	0.114
PLR[a]	59.20 （40.39, 75.58）	49.86 （36.26, 65.88）	0.132
MLR[a]	0.30 （0.24， 0.42）	0.33 （0.25， 0.55）	0.157
SII[a]	323.85 （167.67, 554.95）	365.02 （193.69, 589.38）	0.515
SIRI[a]	1.80 （0.90, 3.19）	2.54 （1.20, 4.63）	0.064
PIV[a]	464.84 （192.32， 1013.39）	664.24 （277.81, 1225.32）	0.151

BPD, bronchopulmonary dysplasia; N, neutrophil count; NLR, neutrophil–lymphocyte ratio; PLR, platelet–lymphocyte ratio; MLR, monocyte–lymphocyte ratio; SII, systemic immune-inflammation index; SIRI, systemic inflammation response index; PIV, pan-immune-inflammation value. [a] Median (quartile) [M (P25, P75)]. * $P < 0.05$.
